# “A quality of heart, of presence, and of really caring”: toward affirmative intersex health communication in Canada

**DOI:** 10.3389/fpubh.2024.1436354

**Published:** 2025-01-06

**Authors:** Terese Knoppers, Angelica Voutsinas, Nicole Palmour, Kaleb Saulnier, Morgan Holmes, Marilou Charron, Hortense Gallois, Narges Jamali, Leslie Ordal, Yann Joly

**Affiliations:** ^1^Centre of Genomics and Policy, Faculty of Medicine and Health Sciences, McGill University, Montreal, QC, Canada; ^2^Social Sciences and Humanities Research Council, Tri-Agency Institutional Programs Secretariat, Ottawa, ON, Canada; ^3^Anthropology Program, Faculty of Arts, Wilfrid Laurier University, Waterloo, ON, Canada; ^4^Faculty of Health Sciences, Simon Fraser University, Burnaby, BC, Canada; ^5^Faculty of Law, McGill University, Montreal, QC, Canada; ^6^Genetic Counseling, School of Allied Health Sciences, College of Health Sciences, Boise State University, Boise, ID, United States

**Keywords:** health communication, intersex, variations in sex characteristics, qualitative, Canada, healthcare practitioners, person-centered care, trauma-informed care

## Abstract

**Introduction:**

This qualitative research study aimed to better understand and help improve the Canadian context for health communication with intersex adults by centering the voices of those directly involved and impacted.

**Methods:**

We conducted 22 semi-structured interviews with intersex individuals (14) and healthcare practitioners (HCPs, 8) from diverse areas of care. Interviews were analyzed via template thematic analysis and filtered through a conceptual lens that brought together agency-based and social-ecological models of health communication.

**Results:**

Findings produced three interlocking axes for change: HCP skills and approaches, structural access to care, and norms and discourses. Participant accounts depict a landscape for intersex health communication where practices are improving, but adverse experiences are still commonplace and intersex individuals cannot assume HCPs will be competent in intersex care. Rather, they utilize a variety of strategies and expend considerable efforts navigating structural gaps and barriers to access affirmative HCPs, who themselves often gained their expertise via individual initiative. Interviewees advocated for HCPs to get a baseline background in intersex care during their medical training, as well as skills in accessible health communication and person-centered and trauma-informed approaches. They also connected broader societal stigma and pathologization to harmful medical practices and called for naturalization and normalization of intersex variations.

**Conclusion:**

This study highlights the need for collaborative efforts across multiple sectors and by multiple stakeholders to drive meaningful change. Findings can help guide HCPs, medical educators, researchers, advocates, and policymakers towards accessible, affirmative, and agency-based care.

## Introduction

Health communication between healthcare providers (HCPs) and patients forms a critical component of positive health outcomes and wellbeing (1–4); “how we talk about a phenomenon affects people in material and life changing ways” (5), p. 512. For people with intersex variations, health communication has historically been beset by stigma, secrecy, pathologization, and harm (5–7). Three decades of advocacy and critique from intersex activists and organizations, medical allies, academics, and international human rights bodies have made important inroads toward medical reforms and there is growing international momentum in the arena of human rights protections for people with intersex variations (8, 9). However, intersex patients navigating and experiencing healthcare systems on the ground attest that the landscape for health communication and care has not appreciably shifted (8, 10). There are implementation gaps between the protective policies and rights that have started to be introduced and actual clinical practice (11). In Canada, like other places around the globe, much work remains to be done toward achieving affirmative health communication as the standard of care for intersex patients (12, 13).

Intersex is an umbrella term for people born with variations in sex characteristics (VSC) that do not fall strictly into binary conceptions of sex. VSC may appear in any combination of a person’s chromosomes, genitals, internal sex organs, hormone production, hormone responsiveness, and secondary sex features (14, 15). There are over 40 medical terms for specific combinations of intersex traits (6). Intersex variations are most frequently identified prenatally, at birth, at puberty, and when trying to conceive (11, 15). While some variations involve specific medical needs and complications, many do not. For a variety of reasons, many people live their whole lives without knowing they have VSC. However, because of advances in genetic testing, more people are finding out about their VSC, regardless of whether they exhibit identifiable characteristics (16).

Individuals use and identify with a variety of language to describe themselves and their bodies, including their specific diagnosis, descriptive terms, and umbrella terms such as intersex variations or diverse sex characteristics (17). However, it is generally asked by the community to avoid the term ‘disorders of sexual development’, currently used in many medical settings, because of its pathologizing connotations (15, 18). In this article we use the umbrella terms intersex, intersex variations, and VSC, as that is the language the international intersex human rights movement (6) and the participants in our study prefer. At the same time, we recognize that terminology will likely continue to evolve.

The intersex community has faced a distinct health communication history of stigma, secrecy, pathologization and harm rooted in medical understandings of intersex variations as inherently problematic. From the mid to late 20th century, the prevailing approach was to treat intersex status as a medical emergency to be ‘fixed’ and concealed (7). Medical management typically entailed a mechanistic sex assignment and ‘normalizing’ medical surgeries and interventions, most often very early on in a person’s life (6). Nondisclosure of intersex status to patients and their families was common. Employing the principle of ‘therapeutic privilege’ as a justification, HCPs would conceal the true reasons for these interventions on the basis that knowing the truth would cause psychological harm (19). Where people were informed, HCPs urged ‘corrective’ medical interventions, frequently omitting other options and overemphasizing incidental health risks (20). These medical norms were tied to broader ideologies around gender, sex, sexuality, and childhood that were crystallizing at the time, as well as to new medical advances and technologies (20, 21). Interventions took place as a misguided effort to foreclose perceived individual, parental and societal distress, and to produce ‘normal’ adults.

However, as many intersex individuals and advocates emphasize, these medical practices cause physical and emotional harm. Physically, medically unnecessary genital and internal surgeries often create health complications such as chronic pain, loss of fertility and sexual sensitivity, incontinence, and the need for regular and/or reparative health interventions (8, 22). Psychologically, secrecy and pathologization impede self-understanding and self-determination, and many intersex people have expressed that they internalized shame, stigma and a sense of isolation (23–25). Mental health consequences such as anxiety, depression and PTSD are also common. Intersex people who were treated in this manner by HCPs frequently avoid medical settings and professionals (57). At the same time, the intersex community has a heightened need for psychosocial and medical support, both because of health problems induced from past medical treatment, and because of the impacts of experiencing social stigma and discrimination (8).

Medical understandings of best practice in health communication began to shift in the 1990s, in no small part due to the influence of on-the-ground intersex advocacy (26). Most notably, the 2006 international *Consensus Statement on Management of Intersex Disorders* highlights the importance of full disclosure and honesty about intersex variations with patients (27). The 2016 revision of the *Statement* further recommended a patient-centered approach (28). These efforts faced criticisms, however, for lacking meaningful input from intersex individuals and for situating medical professionals as the principal authority over intersex bodies and lives (28, 29).

Direct input from the intersex community about best practices in healthcare exists through international intersex organizations. InterACT (US) and Intersex Human Rights Australia have produced best practice guides, both for intersex patients navigating the healthcare system, and for practitioners working within it. These guides centrally emphasize the necessity of informed consent, bodily autonomy, and self-determination (15, 30–32). Other recurring themes include the importance of peer support; the need for accessible, affirmative, and culturally sensitive services; and the benefits of mental health support alongside medical care. Various intersex organizations also exist in Canada. However, at the time of writing, no best practice guides exist for the Canadian context, and community-driven resources and support networks are limited relative to the US (13). Existing Canadian organizations focus on providing ongoing support and advocacy for the intersex community.

The Canadian health communication context for people with intersex variations is not well-established. Canadian healthcare consists of a basic, universal system that is provincially-run, while following certain national principles of coverage set out under the *Canada Health Act* (33). Modalities and resources for healthcare provision vary from province to province (34). Given that Canada is geographically the second largest country in the world, and that its populated areas are spread out, structural inequities of healthcare access and service fall along lines of location, disadvantaging those living in rural and remote communities (35). In addition, Black, Indigenous, 2SLGBTQIA+, disabled, and racialized people, as well as those with a lower socioeconomic status, are structurally disadvantaged in the Canadian healthcare system (35). There is a dearth of empirical literature specifically attending to how having VSC mediates experiences of healthcare in Canada. At the time of writing, there were only two qualitative studies regarding the Canadian healthcare context from the perspective of intersex individuals and no qualitative studies where HCPs discuss best practices in intersex health communication. Holmes (12) conducted a needs assessment of adult intersex Canadians, finding that care should be non-pathologizing, center on the patient as the main stakeholder, facilitate the creation of peer-support networks, and integrate the experiential knowledge of intersex individuals. Sanders et al. (13) found that resources for transitions to new healthcare practitioners across the lifespan are scarce for intersex Canadians, particularly as they aged. They also pointed to a need for medical guidelines tailored to the specific context of VSC and for formalized, Canada-wide intersex networks to foster mental healthcare approaches for intersex individuals. Our study continues this vital conversation.

This research aims to better understand the Canadian context and what is needed toward achieving affirmative communication as the standard of practice by centering the voices of those directly involved and impacted. We conducted 22 semi-structured interviews with intersex individuals and healthcare practitioners from diverse areas of care. Most importantly, this research helps add to the literature perspectives from intersex individuals experiencing health communication in Canada. Qualitative research regarding intersex patient perspectives and lived experience is discursively critical within scholarship on intersex care and practically critical to affirmative advocacy and policy initiatives. This paper additionally contributes to the field in three ways. First, it complements work on the mistreatment of intersex minors and the need to end unnecessary surgeries by highlighting an ongoing need for better health communication practices with intersex adults. Second, while centering intersex participants, it also includes the perspectives of people trained and working in the Canadian healthcare system regarding the current landscape of care and what is needed toward affirming intersex health communication as the status quo. These two groups are not often in dialogue in the literature, and interviewing both contributes to a fuller understanding of the issue. Finally, this research conceptualizes health communication and avenues for its improvement structurally and dynamically, considering what is needed on individual, organizational, institutional, and systemic levels to drive meaningful change. Findings from this study can help guide practitioners and policymakers toward more accessible, affirmative, agency-based care.

## Methods

### Study design

This qualitative engagement research is one component of a larger SSHRC grant initiated by KS and YJ. The grant also involved an international comparative legal and policy review advocating for explicit healthcare protections for intersex people in Canada (9) and the creation of an introductory guide for HCPs on affirmative intersex health communication. A project steering committee including an intersex scholar/advocate and [Montreal and Toronto-based] based affirmative HCPs involved in intersex care (in areas of Psychology, Genetic Counseling, Pediatrics, Endocrinology, Obstetrics and Gynecology) provided guidance on study design and implementation. [YJ]’s research team has backgrounds in intersex advocacy and general 2SLGBTQIA+ advocacy and come to this work from a variety of disciplines, academic and applied, including counseling psychology, sociology, law, political science, human genetics, critical disability theory, gender, and sexuality studies. The study methods lead [TK] has extensive prior qualitative experience. Most of the authors are part of the larger 2SLGBTQIA+ community, and two authors are intersex.

This qualitative study consisted of semi-structured interviews analyzed using template thematic analysis and filtered through a conceptual lens that brings together agency-based and social-ecological models of health communication. Intersex advocates and organizations have emphasized that any research affecting the intersex community should first and foremost center the interests, knowledge, and perspectives of members of this community (36, 37). Keeping this in mind, we made methodological and analytic decisions (38) that foreground intersex voices within the research process and this manuscript. At all stages our emphasis was on ensuring the research was as sensitive, respectful, and useful to the intersex community as possible. Ethical approval for this study was granted by the Research Ethics Board of [McGill University’s Faculty of Medicine and Health Sciences(IRB Study Number A05-B16-19B)].

### Conceptual framework

Our conceptual model draws from and integrates Crocetti et al.’s (8) agency-based model of intersex health and social-ecological models of health communication. Crocetti et al.’s model is itself emergent from qualitative research, as well as intersex and critical disability scholarship. Centrally, agency-based care prioritizes patient self-determination in medical care and interventions, underlining the importance of bodily autonomy and fully informed choice. Crocetti et al.’s model acknowledges that in order to achieve agency-based intersex health care there is both a need to address the sociocultural biases and constructions that currently underpin much medical treatment for people with VSC (such as bodily, sex, and gender norms and pathologization of diversity) as well as a need to actively implement effective care that addresses their embodied health needs (whether variation-related or induced from earlier interventions). We opted to integrate this model with social-ecological models of health communication that attend to the dynamic mutually influential dimensions of interaction between individuals and their environments (39, 40). Social-ecological perspectives emphasize the multiple dimensions (personal attributes, physical, and sociocultural environments), levels (individuals, groups, organizations, institutions), and complexity of human situations (cumulative impact of events over time) (41). Together, these lenses capture the interlocking dimensions in and over which health communication takes place and where an agency-based framework could be realized.

### Recruitment

Recruitment engaged two categories of participants: (1) intersex adults who were familiar with and had received care in the Canadian healthcare system and (2) HCPs likely to work with intersex patients in the Canadian healthcare system (family doctors, nurses, endocrinologists, genetic counselors, OBGYNs, and urologists). First, it was critical to hear and center the experiences, knowledges, needs, and priorities of intersex people regarding healthcare communication in Canada. Second, it was valuable to hear from HCPs trained and working in the Canadian system, and invested in affirming care, regarding the current landscape of intersex care, and where they think the strengths, needs and gaps are.

Understanding that both of our target populations can be hard to reach, recruitment occurred via a combination of convenience, purposive, and snowball sampling and a variety of mediums: email, physical fliers, and social media. Our recruitment write-up and flier circulated via medical listservs, health and social organizations, medical clinics and institutes that provide care to intersex patients, the peer-support networks of some intersex person (IP) interviewees, and the professional networks of some members of our steering committee, research team and some HCP interviewees. Several IP interviewees were recruited via snowball sampling through the recommendation of other interviewed participants. Several HCP interviewees were recruited via purposive sampling to represent a diversity of professions under the umbrella of intersex care. Interested participants contacted [TK] via phone or email and were sent the consent form, additional information about the study, and encouraged to ask any questions they may have. Written informed consent was obtained prior to study enrollment.

### Participants

Respecting intersex participant concerns for privacy and the fact that the size of the intersex population in Canada presents a heightened risk of identifiability, participant information is presented in aggregate to preserve confidentiality. The 14 intersex participants hailed from five provinces (AB, BC, MB, ON, QC), with varying proximity to the health care resources and teams of major urban centers. There was a 60-year age range represented, from early adulthood to senior, and participants spoke about how their relative age affected their histories of care. Seven were in their 20s and 30s, five were middle aged and two were seniors (65+). They employed a diversity of language around their intersex variations, including identifying with the term intersex or using the language of their specific variation. Additionally, four people had trans and/or nonbinary gender identities and brought these forward as further impacting their health communication experiences and needs. Finally, one intersex participant worked in the healthcare system, bringing this additional perspective to their interview.

**Figure 1:**
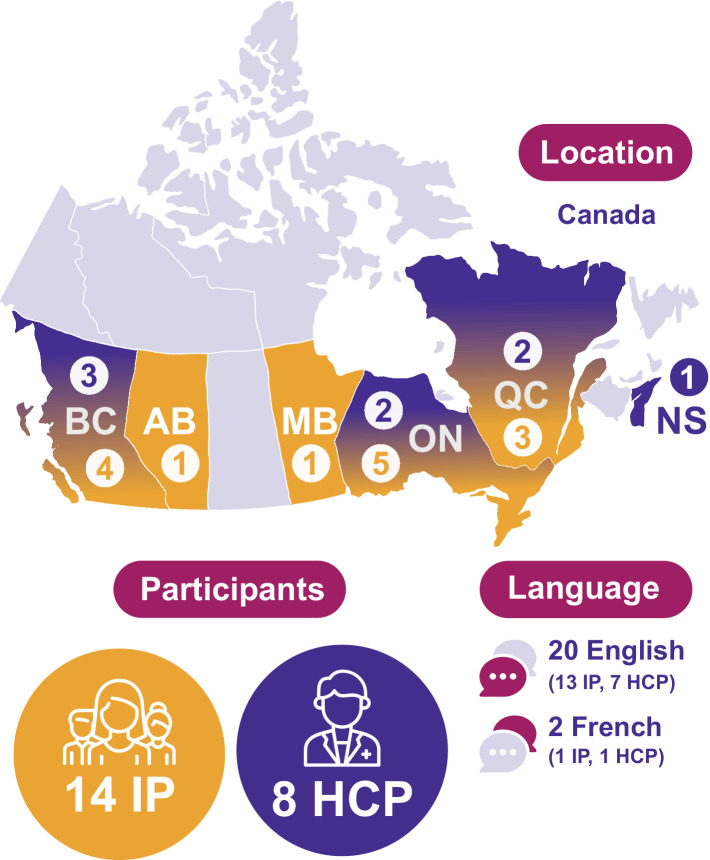
Participant demographics.

The eight HCP participants came from four provinces (BC, NS, ON, and QC). They included two family doctors (FD), one nurse practitioner (NP), two genetic counselors (GC), one medical resident (MR), one OBGYN (OG) and one pediatric endocrinologist (PE). All were early or mid-career, which is significant for the medical norms and best practices at their time of training. HCP participants worked in a variety of areas and specialties. Further relevant to this research was that six out of eight specialize in 2SLGBTQIA+ or trans health as part of their work. HCP interviewees had a range of familiarity with intersex issues, from very little to extensive, and had a range of contact with intersex patients, from none known (*n* = 2), to a few patients (*n* = 4), to consistent regular contact (*n* = 2) (Figure 1).

### Data collection

In-depth semi-structured interviews took place between January 2020 and March 2021. The first author TK did most interviews (13 IP and 6 HCP, all English) and the remaining were done 3 were done by [JH] (IP French), [HG] (HCP French) and [KS] (HCP English). All interviewers had prior experience, and the main interviewer has specialized training in sensitive and trauma-informed interviewing skills.

To maximize accessibility, and due to the interviews occurring alongside the emergence of the COVID-19 pandemic, participants chose between phone and Zoom interviews. Participants were offered the opportunity to receive the question guide before the interviews. Interviews began with discussion about the nature and aims of the research, the research team, and the background of the interviewer. Space was provided at the beginning and end of interviews for participants to ask questions and/or give feedback. [TK and KS] developed the interview questions based on the study aims as well as existing literature and guides by intersex organizations (for the interview guides, see Appendix 1). Questions were designed to be open-ended, trauma-informed, and agency-based. They were worded generally so that participants could choose what and how much to share. The semi-structured format allowed for key topics to be covered and participant-driven concerns and priorities to be raised. Follow-up questions were kept at the depth and breadth interviewees were offering. IP interviews covered experiences and needs within health communication, personal and community knowledge and resourcing, and ideas and hopes for improving the current standard of care. HCP interviews focused on experience with and perspectives on best practices in health communication with intersex patients, related education, training, and gaps within their respective fields, and examples of affirmative care in their own practices. IP interviews lasted between 45 min to an hour-and-a-half in duration. Interviews with HCPs were shorter in duration (20–30 min) in light of study design, and due to their exceptionally busy schedules at the time. All interviewees were digitally recorded with consent. After the interviews, intersex participants were given resource guides created by the team and an honorarium. Accounting for the study aims, specificity of participants recruited, and depth and quality of interview dialogue, the researchers deemed the study’s informational power to be sufficient after 22 interviews and concluded data collection (42). [TK, MC and HG] did all transcriptions.

### Data analysis

Four members of the research team took part in data analysis. We employed template analysis, a form of thematic analysis, for its flexibility, structured approach to coding and its amenability to teamwork and applied research (43, 44). After transcribing and gaining familiarity with the interviews through repeated readings and note-taking, [TK and HG] coded three interviews each on the QSR software NVivo 1.5. [TK] bottom-lined IP coding and [MC] led HCP coding. Codes were identified inductively and at a semantic level so that they would be strongly linked to the data itself (38). Then, [TK and HG] met and collaborated on the creation of an initial template on Microsoft Excel, bringing IP and HCP codes together, clustering them into meaningful groups and organizing them hierarchically (43). The template was iteratively revised and refined based on sets of 4–6 interviews. [TK, HG, and MC] traded off coding and validation roles, meeting up to discuss after each set. Special attention was paid to the layers and operations of health communication in participant accounts, as well as to where agency was enabled, enacted, and elided. Depth was emphasized, with coding going into four sublevels. As the research concerned an area lacking qualitative data, the overall goal for the final template was to represent salient codes across participant interviews and groups (38).

In preparation for presentation in a narrative format, [TK] used mind mapping to reorganize, combine and synthesize the coded categories into themes and subthemes central to the research aims and in light of the conceptual framework (44). Mind mapping allowed for further conceptualization and exploration of the relationships between codes, themes, and subthemes outside of the linear structure of the template (38, 44). Then, as a final validation measure, [TK and AV] re-read the interviews with the thematic map on hand and made some adjustments to subthemes. Themes and subthemes are presented in the Results section, along with a combination of shorter and extended quotes to maximize participant voice. Supporting quotes have been de-identified, cleaned of fillers and dysfluencies, and translated from French to English where relevant (45).

## Results

### The landscape of care

This first section covers participant accounts regarding the background context and factors at work before the healthcare encounter; before individuals with VSC walk into the room to meet with a healthcare practitioner in Canada. Four related structural threads emerged from interviews: (1) lack of consistent HCP training to work with intersex patients, (2) strategies intersex patients employ to try and access effective care, (3) compounding structural access factors, and (4) self-resourcing and underuse of medical services.

#### Lack of coverage of intersex issues in medical programs

Participants generally painted a landscape where there are some HCPs who, by virtue of their specialization, or individual professional efforts, have training in intersex care, but where coverage in Canadian medical training programs is typically cursory, circumstantial, or absent. For example: *“The learning I’ve had has been through my own work and attending conferences and workshops, not so much from medical school or training”* (HCP6, FD); *“We received one hour on intersex issues out of a year and a half of classes in medical school”* (HCP2, MR), and HCP1 (NP):

*I sought it out myself. There is a very big gap. I have been a nurse for 7 years, so I’m hoping things have shifted and changed. I know as far as medical students go, they get a little more training and opportunity to provide care, but even that is very minimal… It depends on what university you are attending, the instructor you have, and what clinical rotations you get access to. It’s a practice that should be accessible: you should be able to go to any family physician and anticipate that you will receive care as an intersex person. But that is not necessarily the case, the reality is that people are discriminated against a lot and there are an incredible and alarming amount of barriers for folks.*

Endocrinologists, gynecologists, urologists and genetic counselors were named as professions more likely to receive basic medical training in this regard: *“During our training as a general endocrinologist, in clinics and at the more academic level; we have courses on that and then we obviously learn by following our families. I wanted to go further so that’s why I went to do an extra year, but otherwise yes, we have basic training during our residency”* (HCP8, PE). This variation in familiarity and preparedness across HCPs in Canada has implications for intersex patients: *“Depending on your entry point into medicine, you are either going to get a lot of support or you are not going to get any support at all”* (HCP4, GC).

#### Screening strategies for finding competent care

Intersex interviewees were keenly aware that many of the HCPs they were going to encounter would not be competent in intersex care. In the absence of a centralized directory or hub for intersex affirmative providers in Canada, patients themselves end up doing the work to find competent providers:

*There is not really a central space where you can be like, ‘I’m going to start there!’, you have to think about where you are and what’s around you…then keep branching out as you can. That took a lot of trial and error. I called four or five different offices that had someone with trans or intersex care in their description and it was like “yeah, we do not do that anymore, or he retired, or our patient load is full.” So, a lot of denials and rejections* (IP7).

IPs had a variety of strategies they employed to maximize contact with prepared HCPs, minimize contact with unprepared HCPs, and within the latter, minimize adverse experiences. IP8: “*I’ve just heard horror stories of people encountering really poor support within the Canadian healthcare system, and so my advice would be to try and find somebody who knows something about it…. who is educated and aware of these things.”* Participant strategies included finding HCPs via recommendations from other intersex people, researching and screening HCPs online via reviews, reading the healthcare environment for cues, self-advocacy, and bringing a support person. Multiple interviewees brought up specific affirmative HCPs in Canada who people find by word of mouth:

*There is one doctor in particular that I want to mention who has been the doctor for several of my AIS [Androgen Insensitivity Syndrome] friends. She is just so good. She listens, she is supportive…They’ve had really positive experiences of being diagnosed, of being helped through the whole process* (IP5).

For those HCPs found via the internet, participants described the initial visit to a new HCP as ‘nerve-wracking’ “*because you do not know how you’ll be treated by the doctor”* (IP11). Some read the environment for cues: “*There’s some clinics where they put up stickers saying: “all people welcome” and that kind of thing. I look for those because I know I’ll likely have a better experience”* (IP1). Non-inclusive environments had a negative impact: *“I do not feel included in the forms, even in the speech at times”* (IP4). Understanding this, a couple of the HCP participants emphasized the importance of communicating a welcoming healthcare environment, physically and administratively: “*Making sure that all of our forms are as inclusive to as many different people as possible who walk through our doors and that we are providing a welcoming inclusive environment”* (HCP4, GC).

In order to best facilitate positive healthcare experiences, IPs spoke about the importance of self-advocacy: *“I would say the main thing is advocating for yourself if you feel that your needs in healthcare aren’t being met”* (IP9). They emphasized that individuals with VSC should feel comfortable communicating their questions: *“It’s perfectly okay to be like, hey, hold on, can you please explain this definition?”* (IP4); and needs with their HCPs: *“They will not know what you are going through. They do not know what you need. I feel like stating what you need, how you need it, and what your preferences are really helps the medical professional be able to help you”* (IP1). Many IPs also expressed that they ended up actively educating their HCPs and/or managing stigma themselves: *“I feel like I carry a lot of the burden of the conversation…Socially, I have to let them know it’s okay, calm down their uncomfortableness. So, it’s the education component and it’s the ‘hey I’m just a girl, I’m a mom, I’ve got kids’”* (IP14). Finally, some participants noted that bringing a support person to appointments can be helpful. Overall, these strategies consume considerable energy and time: *“I’m going to have to go through it again. It takes a lot of energy from me to feel the motivation”* (IP10).

#### Compounding structural access factors

In addition to the lack of training of HCPs on intersex needs and issues three compounding structural factors in Canada came up: age, location, and the intersection between intersex and 2SLGBTQIA+ care. First, children’s services were described as better resourced, more personal, and more comprehensive than adult services: *“The level of care just wasn’t the same”* (IP8). Interviewees shared that the transition to adult services can be difficult and disjointed. IP12:

*I was seeing the same healthcare practitioner for a very long time. Until I turned 18, then I had to stop seeing them. Since then, I have not had regular visits with a healthcare practitioner… that’s something that I’ve heard echoed back to me; that it’s really easy to get consistent care with the same doctor until you are an adult. It’s a lot more challenging when you are an adult*.

One participant expressed how the disjuncture between pediatric and adult services also functions to create a disjuncture in HCP responsibility: *“Nobody is actually there to be held accountable for removing my gonads at seven years old and to help me figure out my hormones for the rest of my life”* (IP14).

The second compounding structural factor that repeatedly arose was the impact of location on access to HCPs. Within Canada’s vast geography, intersex participants not located in major cities described traveling considerable distances to gain access to the medical services they need. Access issues impacting everyone living rurally or remotely from large cities were exacerbated for intersex interviewees. IP14 again:

*I have to go all the way to [a major city]to get testosterone. There’s no reason my local doctor could not prescribe me hormones except for the fact that he’s scared. But nope, I have to see an endocrinologist and it’s a seven hour-drive. This is to get a prescription for something I’ve been on for years*.

Travel requires financial resources (via expenses and lost work), further impacting access: *“Socioeconomic circumstances also impact access to care, as wrong or right as that might be, we know that that’s a huge factor”* (HCP5, GC).

The third factor interviewees raised was the relationship between intersex care and the 2SLGBTQIA+ health resources that exist throughout Canada. Some intersex interviewees sought out providers specialized in 2SLGBTQIA+ care because of the ‘I’ and/or because they further identified with other identity categories under the acronym. The hope was that clinicians working under that umbrella would have competency, or at least be supportive and non-pathologizing regarding VSC. Indeed, several HCPs spoke to this: *“There’s an overlap just by the nature and interest and expertise people have”* (HCP7, OG). However, a few intersex participants voiced that even though they experienced 2SLGBTQIA+ contexts as relatively more affirmative, the ‘I’ in the umbrella could be better attended to and resourced: *“We are a minority community in the LGBTQ long list; we are a minority of the minority. So, if we could have more representation there, and then be involved in the education of healthcare practitioners as well”* (IP1). This sentiment was echoed by a couple of the HCP participants working in 2SLGBTQIA+ care who themselves felt underprepared for intersex patients and wished for more training: *“I do not specifically have the tools for if I was working with someone who is intersex. I do not particularly know how I might change that”* (HCP6, FD).

#### Self-resourcing and underuse of medical services

The final ways in which intersex interviewees exercised agency were to learn as much as they could about their own diagnosis and health needs and to find intersex community. Significantly, many responses to the interview question “Do you have any advice for intersex people navigating the healthcare system?” were in this vein. For example:

*There are so many systemic issues and so much learning that needs to be done in the healthcare system that you should not take it at face value, there’s just so much going on. So, whatever is presented to you at the time, continue to explore that…Do not sit in the feeling alone, try to gain a community in whichever way you can. It has been really wonderful to hear from other folks about their experiences – it has been life changing* (IP4).

Put differently, the advice for how to navigate the healthcare system anticipated unprepared and/or adverse health communication as the status quo.

Some participants ended up delaying or avoiding care altogether. IP10: *“I avoided, and I avoid still. I avoid my healthcare.”* Underuse of services stemmed both from difficulties accessing supportive HCPs and from negative or traumatic healthcare experiences: *“To be honest, from that last time, I became very skeptical about accessing healthcare. I thought: ‘what’s the use if I’m going to have to hear opinions about my body?’, how I should be, you know?”* (IP13). Interviewees thus spoke of underuse of healthcare services as a systemic issue: “*As long as there is a lack of therapeutic trust, there’s going to be a segment of the intersex population that will not have adequate access to health, who will refuse to go see a physician”* (IP2).

### Directions for improved health communication

Given the landscape of care in Canada, this second section is concerned with participant feedback regarding what is wanted and needed to achieve accessible, affirmative, agency-based health communication as the standard of practice. Interviewees advocated for four standards for improved health communication: for HCPs to have a baseline background in intersex care, skills in accessible and relevant health communication, a person-centered approach, and a trauma-informed lens.

#### Baseline background in intersex care

In terms of training needed, interviewees agreed that all HCPs should have a baseline readiness to work with intersex patients. This included introductory medical education about intersex variations and common indicators that they may be present in a patient: *“Knowing what the things are that might cue that there may be an underlying [variation]. You know, a delayed puberty, the absence of menstruation,* etc. *Just even having it on their radar as something that could be going on”* (HCP7, OG). Central to background competence was a depathologizing lens: *“I think it should be taught in school that being intersex is just a normal variation and not a pathology that needs to be corrected”* (IP13); and the idea that sex can be understood as a spectrum, rather than a binary:

*I would love for the curriculum for healthcare practitioners to include the understanding that sex is a spectrum…because those who lean on binaries really have an issue with how they come to understand intersex identities. At times there can be confusion or just like “this is something that should not be there for you, because you are female or male,” but that’s not true and it’s not helpful for folks* (IP4).

Several participants urged that this background knowledge is particularly important for family doctors as they are central figures in the healthcare system: *“They are at the front of all this and they are people that you keep going back to”* (IP5).

Alongside baseline medical knowledge, interviewees wanted HCPs to learn that intersex care is an area where psychosocial support is essential: “*It’s really important to have both the medical and psychological aspects”* (HCP8, PE). They emphasized the need for HCPs to learn to either provide psychosocial support or to make the appropriate referrals. Indeed, this was seen as a part of de-pathologizing VSC: *“Medicine should restrict itself to only performing interventions that are linked to health difficulties, and then for everything that’s tied to bodily differences and that does not cause any health problems, have non-pathologizing psychosocial support”* (IP2). Participants voiced that there are life periods and areas where patients with VSC may need more support, including when they first learn of their variation, around any related health or bodily implications, and around their medical options. Unfortunately, access to psychosocial support was not always part of IPs’ experience. Finally, interviewees wanted HCPs to understand the importance of peer support for individuals with VSC, and the role HCPs could play therein: “*I think the important thing is to connect them with whatever resources are available…local or larger scale support groups and that type of thing”* (HCP7, OG). A couple of the intersex interviewees had been referred to community support via their doctor and spoke to its impact and meaning for them: *“She referred me to the group, which has been hugely helpful. I’m not so involved now, but in the early years it gave me a lot of comfort”* (IP6).

#### Accessible and relevant information delivery

Participants continually highlighted the importance of relevance and accessibility in health communication. Relevance came up in two ways. First, intersex interviewees asked that their VSC come into the discussion only when medically relevant: *“Even if it’s something like a small checkup or prescription or test that’s completely unrelated to the reason of my visit, it comes up almost every time”* (IP12). Second, they asked that the health information they are given be specific to their VSC. A few participants shared experiences of receiving information about other VSC, for example IP8: *“I remember talking to my mom and being like ‘Oh mom, it says here that an egg can be implanted into my uterus!’ and she’d be like, ‘Oh no, you do not actually have one of those…’.”*

Interviewees additionally discussed how HCPs should be aware that the language they use to discuss intersex variations can pathologize their patients, and that it is important to use current terminology: *“Hearing ‘disorder of sexual development,’ or about having a ‘condition’ or a ‘syndrome’…it’s language that perpetuates there’s something wrong with being intersex”* (IP12). Multiple intersex interviewees expressed feeling unsupported and unseen when their HCP used highly medicalized language to discuss their VSC:

*When a bunch of medical terms are thrown at you and you are just sort of like a case file… you feel the opposite of supported. When you think of it, it’s these medical terms…but it’s not just terms, it’s experiences too. A human experience* (IP4).

HCPs agreed with the need to avoid medical jargon with their patients: *“If I have to use medical vocabulary, I do my best to explain what I’m talking about just so that they feel like they have some agency within their own health”* (HCP1, NP). They spoke about how accessible language looks different for every patient and emphasized that HCPs should be meeting patients at their level: *“There’s a lot of variation in how much health and scientific teaching people get in their education”* (HCP7, OG).

Finally, participants spoke about the pacing and means of information delivery as part of accessibility. Interviewees highlighted that receiving information about VSC can be an overwhelming experience, particularly when not given time to process. Several HCPs discussed titrating information as an effective strategy:

*There’s certainly patients where we have gone over a lot of information in one session. And others where we have given them the results, given them a few tidbits, answered a couple of questions, and left it at that. Then booked another appointment to go over things in more detail once they have had some time to digest those results* (HCP5, GC).

They further shared that it can be helpful to combine verbal information with physical or web-based resources that patients can access on their own timeline.

#### Person-centered care

In terms of approach, HCPs and IPs alike advocated for person-centered care. Centrally, intersex interviewees wanted HCPs to approach them respectfully, as multi-faceted individuals, and to listen to their experiences and concerns: “*The thing is that everyone has a different story on how they see themselves, what their experience is and where they are at in their journey…if you have met one intersex person, you have met one intersex person”* (IP14). Being treated with warmth, dignity, and compassion made a significant difference—“*A doctor who listens to people, who supports people, that empathizes, has compassion…to me this is important”* (IP5)—and IPs appreciated when their doctors took the time to get to know them. HCPs reiterated the importance of getting to know their patients, adding that it allows HCPs to support their patients’ care most effectively: *“Everything from a family’s socioeconomic status, to how they interact with one another, to their life circumstances”* (HCP5, GC).

Unfortunately, nearly every intersex participant had had experiences where they did not feel seen or even related to as a human being: *“You can feel like an alien specimen in a way”* (IP7). They attributed this treatment to both individual and systemic factors: *“Some physicians have that god complex”* (IP11); and:

*There are parts where people get so far into their thinking mode that they bypass all the heart, the realness, the being there and being present. I think it’s a real challenge to actually be present to every single person coming in for a 20-min visit. You’re stuck in a room, you are like a rotating door and I can see a lot of people get drained from it. I think listening comes from the heart and I think that’s a big-ticket item that’s missing from some healthcare professionals: a quality of heart, of presence, and of really caring* (IP10).

IPs spoke poignantly to the human impacts of a more paternalistic and pathologizing approach:

*You know you can really hurt people, you can leave them damaged, unintentionally maybe, but you can do it. If you do not really support them or make them feel like an okay person because this is something that’s ‘wrong’.* Versus *‘we can deal with this’ or ‘we are going to go through this together’, those things make all the difference* (IP5).

Recognizing this, many of the HCPs in this study described working to ensure their patients felt seen and supported: “*We try and create a safe environment…make sure families feel like they have everything they need”* (HCP4, GC).

Shared decision-making also came up as a component of person-centered care. Interviewees articulated an ideal scenario where HCPs and patients make health decisions together; HCPs are honest and transparent, informed consent is thorough and ongoing, and IPs are supported to consider different options and achieve informed preferences: *“Someone who will not decide for me, but rather will work with me in making certain decisions once I have all the information that I need”* (IP13). IPs spoke of these experiences among their most positive ones: *“She was a practitioner that I went to as we were trying to explore different hormone replacements. She actually started learning about it. I was able to talk to her, able to converse about what was happening for me and what I wanted to go forward with”* (IP10). Similarly, most HCP interviewees tried to facilitate shared decision-making: *“Make sure not to be condescending, to really be on the same level with the families, and then to really include them as much as possible in the decision-making process, to make them understand they really are full team members”* (HCP8, PE).

Multiple IPs had experiences of HCPs not properly disclosing their VSC to them: “*I knew intuitively that they were not telling me the truth. It just did not make sense, as little as I knew at that time…And there were no answers. If you asked questions, they would change the subject”* (IP5). HCP8 (PE) similarly raised the issue of lack of transparency: *“In the past there was a lot of secrecy, a lot of secret diagnoses- not all information was disclosed to families and to young people. It really created gaps in communication and it broke the bond of trust with medical professionals”* Some interviewees also expressed not being made aware that they had options before undergoing medical interventions: *“It would have been nice to talk a bit more about my options. Honestly, until recently I always thought that it was fatal if I did not take testosterone. I did not know that an option would have been to not take anything”* (IP3). Not being given options was a particular issue when making decisions about surgery: “*I have gone to see a doctor and had them push surgery for me, only to find out that it wasn’t necessary, and I did not get it, but that is a very common thing that intersex people face”* (IP12).

#### Trauma-informed care

Most IP interviewees, and all who were middle-aged or senior, shared negative medical experiences in Canada. A third of the intersex participants explicitly used the language of trauma. Adverse health communication experiences included nondisclosure or highly insensitive disclosure of their VSC, overstated medical risks, particularly around cancer, pressure to have surgeries, fixation on and questions around VSC when not medically relevant, not being listened to or given space to ask questions, and unwanted and/or inaccurate communication around sexual intercourse, their attractiveness and bodies, including communicating as if they were not in the room. They articulated that pathologizing health communication made them feel alone, undesirable and like something was fundamentally wrong with them. Interviewees described profound physical and psychological impacts of these experiences. Two poignant examples:

*I’ve never really stabilized. To this day. I wear an estrogen patch in order to keep from having severe hot flashes, because I can still have them if I go off estrogen. It keeps them milder, but I still have the occasional small one each day. So, I’m reminded every single day of what happened, like it or not, and why it happened, and what I am* (IP5) and IP10:

*There are things happening that are worthy of some major medical liability and causing some major major trauma for people who then spend years afterwards in therapy, years trying to heal themselves from this and start going forward. There are people walking around with death wishes…That’s why Canada needs to stand up and become what it’s supposed to be…it’s supposed to be on the forefront of human rights.*

For participants IPs, past experiences underline how they now interface with healthcare:

*I’ve been left with a massive distrust of anything medical, even to the point where we are now going to discuss my parents’ care, and I distrust people who I have to get involved with. I’m gearing up, so what’s been fostered in me is to fight. Fight or flight, and that’s PTSD in my opinion* (IP12).

Given this background context, interviewees discussed the need for trauma-informed health communication. Most centrally, they emphasized that it is critical that HCPs be aware of this history and that patients with VSC they encounter may have had past medical trauma: “*People deserve to have you listen to the painful things that they are saying about past treatment in medical communities. So, a knowledge of the historical harms and historical workings of the medical establishment is important”* (IP14). Several referenced that this has not necessarily been the case in the relations between the intersex and healthcare practitioner communities:

*To learn the way in which physicians have reacted to intersex people who have publicly shared their criticism or all the damage from the non-consented interventions that had been done to them. I wasn’t expecting that. I wasn’t expecting that physicians would react with such resentment, with such denial, with such contempt…it was really difficult to get closure or to enter a healing process* (IP2).

At the same time, most IPs and HCPs in this study were hopeful: *The past has caused so much trauma. It is changing, but it’s like righting a ship, a massive massive ship…Yes, so I think it’s good that this is happening, it’s good that it’s coming out into the open* (IP10). IPs spoke about how healing it felt to be affirmed:

*The doctor that was in charge of the medical research center when I was there…after I learned all that I’ve learned, I confronted him, and told him how my experience had been so difficult and damaging and harmful. I did that and he apologized and he spent a couple of hours with me. And that was one of the best things I could have done. Having an apology and having him listen to me and say ‘okay what we did was not the right way to do things’. It made a big difference for me* (IP5).

Several HCPs referenced witnessing the ongoing impacts of past medical standards of early surgeries and nondisclosure in their own practice, particularly on patient trust and self-knowledge: *“I’ve had situations where there is a lot of confusion about exactly what has been done and when to the patient”* (HCP3, FD). Some shared how they integrate a trauma-informed lens into health communication. HCP 1 (NP) talked about being attuned to when trauma can surface: *“Because of what we talk about and peoples’ intersections of oppression and their histories of engaging with* var*ious layers of healthcare systems…every interaction can be emotional in the sense that it can cause difficult feelings to come up or could trigger their past traumas.”* HCP6 (FD) spoke to the importance of not personalizing trauma responses and of ongoing consent:

*I think my approach is really just to be very open, very receptive, understand that everyone is coming in with their own life experiences and that if they are having a hard day or not immediately super warm or forthcoming with me, that might have something to do with me, but largely might have something to do with how they experienced healthcare in the past and what they have had to worry or anticipate might happen…The other thing is just consent-based practice all the time. If I’m asking personal questions, being prepared to explain to someone why I’m asking certain questions. Then, definitely with physical exams, making sure I’m explaining what I’m doing and why, and giving people the option to decline or postpone to another day if they feel uncomfortable, or to bring someone with them if that feels more comfortable.*

Overall, trauma-informed practice was articulated as critical to affirmative intersex care.

### The need for discursive shifts

The third and final section covers interview content on the norms around VSC that circulate in broader society and how they need to change. Participants reflected on how broader societal norms and discourses concerning VSCs impact medical understandings and care, affect people’s lives more generally, and limit culture by eclipsing a type of diversity.

#### Naturalizing and normalizing intersex variations

Beyond the walls of healthcare institutions, participants spoke to the need to shift how people understand and think about VSC more broadly: *“Once society is accepting of what’s in between, the whole spectrum of male and female and the whole grey area, then things will change. Once society says it’s okay to talk about this”* (IP10). Medical norms were articulated as contributing to, but also circulating within, broader societal understandings of sex and gender. For example:

*There are a lot of systems in place in the medical system and also everywhere in the world that are so extremely binary in terms of gender and sex. It’s the first thing we say about a patient… it’s just very binary. It excludes people and in every way it’s a fudging of the truth. It’s make-believe that we even say that. I’m sure being faced with that all the time and in every system that you are part of would be extremely challenging* (HCP2, MR).

Interviewees called for the naturalization and normalization of VSC as an aspect of human diversity: *“Beyond just knowing about intersex people, having the point of view that intersex variations are not something that need to be fixed. They are something that exist in a lot of people, and that should be celebrated and not shamed”* (IP12). Intersex and HCP participants alike articulated ways they try to contribute to broader shifts. On the part of IPs, doing advocacy, giving talks to various audiences (including HCP students) and participation in affirmative research were all mentioned: *“I think it’s really empowering for intersex people like myself to share their story. Hence what I’m doing right now. I’ve done little talks just to broaden people’s minds because it helps to normalize it- talking openly about it”* (IP3). HCPs talked about normalizing VSC within their practice:*“[I tell them] it’s just another piece of information that we know about you, that you have had since birth, and it does not change who you are as a person, it just so happens that we know about this about you”* (HCP5, GC); and via trainings for other HCPs:

*We put together a 1–2 hour workshop on how to specifically care for people who identify as LGBTQ+ and genetic counseling. We took it as a kind of roadshow. We were going to different institutions and different conferences and giving that lecture on different platforms or as posters, to get the word out there that this is one of the tools in our genetic counseling toolbox that we need, and to fill in that gap in people’s education* (HCP4, GC).

Throughout their interviews, people with VSC also shared the value of those medical professionals they had known as allies; who they trusted and respected and felt went above and beyond: “*There’s also a seat for doctors to become allies”* (IP14). IP5:

*I had a wonderful endocrinologist, who has passed away now. He was very good and he was someone who used to ask me to come talk to his residents. He was a really special person. There was another person at [local medical school] who would arrange for these AIS groups we had to come speak with the medical students about our experiences with the medical profession. It was all part of a course he was teaching on how to be a better doctor and listen to people, be concerned and receptive, that kind of thing. He really was a good person.*

Finally, some interviewees poignantly expressed ways that they experience being intersex as empowering, as adding value and perspective to their lives: “*I think it actually makes my life a little bit more interesting, getting the best of both worlds”* (IP1). These accounts take naturalizing and normalizing a step further, to actively appreciating what being intersex brings and contributes to the human experience. IP12:

*I’d say that being intersex is very central to me as a person. I think that it’s kind of shaped me, made me who I am today. I’ll always be grateful for what it has brought me, growing up with that lens on the world, being able to see, not only black and white, but also those colors of grey, in between. It sounds cliché, but I genuinely think I would not change anything and I’m grateful for that.*

## Discussion

**Figure 2:**
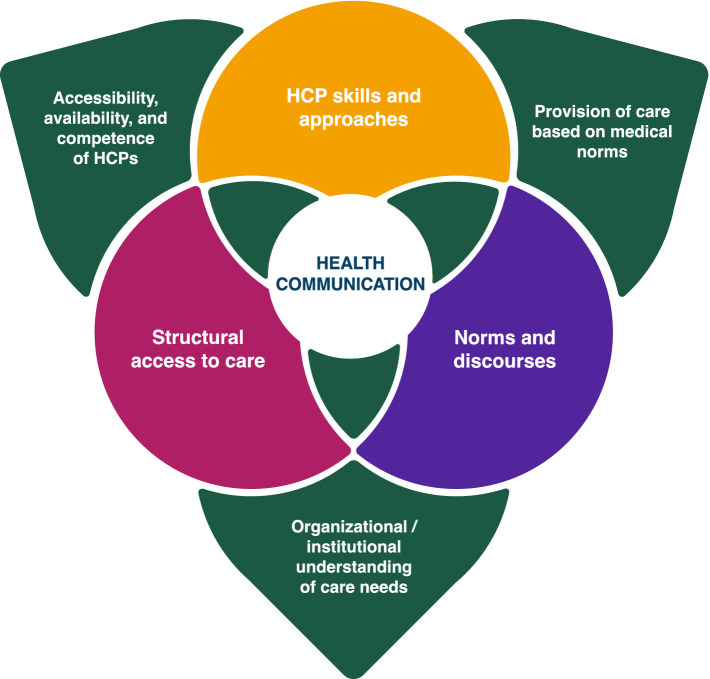
Interlocking axes for change.

Filtering our results through our conceptual model, Figure 2 illustrates three interlocking axes for change in Canada to work toward accessible, affirmative, agency-based health communication as the status quo. These axes encompass personal, interpersonal, community, institutional, structural, and discursive levels of how VSC circulate and materialize (or not) into healthcare access and provision.

### Improving HCP communication

Intersex patient and healthcare practitioner accounts indicate the need to improve standards of intersex health communication in Canada and to address the ongoing adverse impacts of past care standards in many patients’ lives. IP experiences in particular underscore how the burden of poor health communication falls on the patient. Our findings echo that of Crocetti et al. (8), numerous other scholars, and best practice guidelines that advocate for a model of intersex care that centrally promotes patient agency, includes the provision of psychosocial support, and in which medical care is reserved for medical situations and needs (5, 12, 13, 29–31).

As participant accounts show, communication begins with the healthcare environment; the overlay of social and environmental factors also arbitrate agency in healthcare access (8, 46). IPs shared that given their past experiences, they read healthcare environments for safety cues. Inclusive intake forms and signage were taken as encouraging. Inclusiveness and safety can be further promoted via training staff about VSC and empathic communication skills and having office and reception areas where private information can be shared discreetly.

IP interviews attest to the substantial work they do, not only to access healthcare but within communication with their HCPs. Our findings resonate with other studies and writing on interpersonal health communication for patients with VSC, for instance on stigma and stereotypes within care (8) and on patients having to educate their HCPs (47). It is important for HCPs to understand what many IPs metaphorically ‘bring into the room’ with them in terms of the history of care and their past experiences, as well as the psycho-emotional impacts of HCP communication. There were explicit incidents of harm throughout participant accounts, but unpreparedness when engaging in care with someone with VSC can also lead to harm that may be initially less obvious (12, 29). Thus, the baseline readiness for intersex care as well as person-centered and trauma-informed approaches advocated for throughout participant interviews were not merely reparative but protective.

Knowledge and experiences from the IPs and HCPs in this study also illustrate how to implement effective affirmative care. Recommendations that the initial approach to patients be open, respectful, collaborative, concerned with wellbeing and not focused on VSC unless the motivation for care warranted it exemplifies agency-based healthcare (8). Attentive listening, engaging patient concerns, a depathologizing lens, and whole-person care have been noted as essential to trust and safety (5, 12, 29). Tailored and titrated information delivery in terms of quantity, patient context and readiness, and specific VSC, are in line with recommendations in other Canadian literature (13). Similarly, the need to facilitate access to psychosocial care and peer support has been emphasized by qualitative studies in Canada and the United States (12, 13, 29, 48). Finally, trauma-informed approaches, including the use of sensitive language, attention to non-verbal cues, and foregrounding of explicit consent around medical exams and processes have increasingly been recognized as essential to HCP repertoire (3, 23, 29, 49).

Notably these approaches for affirmative health communication—interpersonal, organizational/environmental and community—are rooted in and reflect social-ecological understandings of the mutually influential layers of health communication and how agency can be experienced and enabled therein (39, 46). Ultimately, the four competencies that participants asked for toward improving care are copacetic with training and direction already being implemented for HCPs more generally, and with preparedness to work with populations from other marginalized social locations. Training and continued education opportunities catered to the needs of patients with VSC is readily achievable.

### Addressing structural access barriers and gaps

In order to facilitate affirmative health communication, structural barriers and gaps impeding access to care must be addressed so that IPs are able to connect with HCPs in the first place. By removing barriers and better resourcing intersex healthcare, IPs are given the opportunity to experience agency-based health communication, healthcare centers to provide it, and HCPs to practice it. This research contributes the access dimension to Crocetti et al.’s model (8) and helps fill a respective gap in the broader literature.

Interviewees spoke of three compounding access barriers to intersex care in Canada. First, how adult services are not as well-resourced, or comprehensive, as children’s services (50) and the subsequent difficulties of transitioning to adult services (13). Individual HCPs can support IPs after they age out of children’s services by helping them find adult care (50), following adult patients long-term when possible, and being responsive to the extra supports needed for other transitions that happen as part of the life course, such as adapting to new health information or aging (13). Intersex participants not located in major cities, where specialized intersex care tends to be, listed location as a second compounding barrier to care (51). Location-based inequities are increased for IPs with less time, capacity, and resources to travel for care (25). Establishing strong telehealth options would help IPs be able to access adequate intersex healthcare regardless of where they live (52). Finally, given the dearth of intersex-specific HCPs, intersex interviewees reported seeking out providers in 2SLGBTQIA+ health. Combined with the fact that many of the HCP interviewees work in 2SLGBTQIA+ health, it is evident that this has become an access point to intersex participants care in Canada. While 2SLGBTQIA+ spaces may be a good venue, IPs emphasized the need to ensure that intersex healthcare is well-resourced, not conflated with trans or queer healthcare (53), and that this not be the only venue for intersex care to accommodate those people not comfortable under this umbrella.

HCPs reported varying levels of familiarity with intersex health given the lack of coverage on the topic in Canadian medical training programs. Studies from the United States report a similar phenomenon (29, 54). While some HCPs have been making individual efforts to self-educate, this is an individual solution to a structural problem. IPs consequently experienced inequitable standards of care and described going into healthcare settings assuming a lack of HCP preparedness (47). The burden fell on IPs to manage inequities in healthcare access and provision via screening strategies, self-resourcing, or turning to their communities instead of HCPs and healthcare services. While these constitute practices of agency and resilience within a healthcare system that is failing them, they also demonstrate the need to remove barriers to quality intersex care in Canada. Including intersex care as a part of medical training for all HCPs in Canada is necessary to address this problem (54). Establishing a centralized Canadian directory of affirmative HCPs could further help reduce the work IPs have been doing to access adequate care.

Within the current healthcare landscape, both HCP and IP interviewees demonstrated individual initiative-taking to mitigate structural resource gaps and access barriers. While these actions are resourceful and significant, structural change is necessary.

### Toward affirmative norms

Communication from healthcare practitioners influences and is influenced by sociocultural norms concerning health and bodily differences. Crocetti et al. (8) outline two interrelated norms that produce and reproduce the lack of agency-based healthcare for people with VSC: binary essentialist understandings of sex and gender and the ideas about acceptable ‘male’ and ‘female’ bodies. Both are misconflated with healthy development and result in medical attempts to ‘normalize’ people along these lines (55). Both locate the problem in individual rather than larger societal forces that denaturalize and attempt to eliminate difference.

The fact that all intersex interviewees in this study navigated stigma and pathologization stemming from these norms to some extent, including as adults, and regardless of their personal identities and medical history, underscores their perniciousness and the need to change them. Notably, those with more supportive families and communities and those from more recent generations named more protective factors in navigating, resisting, and upending their impacts.

Intersex and critical theorists have repeatedly spoken about the need to disentangle sex and gender and value the multiplicity of human diversity therein as contributing to humanity (7, 55). This research contributes a qualitative lens to this conversation on norms and a social-ecological lens to the conversation on intersex health communication. Affirming agency-based health communication is necessary but not sufficient to ending the pathologization and stigmatization of VSC and its concomitant harm. Once societal norms shift, measures of health can as well. For example, the presence of supportive family, community, and health professionals, a positive relationship to self, autonomy and self-determination can be prioritized as important to wellbeing (5).

Participants understood and articulated medical and larger societal norms around sex, gender, and sex variations, as operating in concert, as changing over time and as being influenced by sociopolitical and structural factors. They made explicit and concerted efforts to help shift the discourse from pathologization to naturalization and normalization of VSC. For IPs, this, more often than not, necessitated internal self-resourcing and repair work via intersex community and psychosocial support. It also encompassed external advocacy work, talks for HCPs, and participation in proactive research. On the part of HCPs, the work was about becoming affirmative care providers for their intersex patients and in educating other HCPs, including bringing people with VSC to speak to HCP audiences. Such individual efforts can cumulatively contribute to broader shifts. Ultimately, there are additional important actors and forces at play in any renegotiation of meanings and knowledge around intersex and sex diversity (5, 56). Indeed, naturalizing and normalizing intersex variations will require a multi-level, multi-sectoral effort, centering the knowledge, needs, and agency of people with VSC and accomplished alongside supportive HCPs, human rights and advocacy groups, academics, lawyers, politicians, and policymakers.

### Limitations and future research

Study participation was self-selected and should not be taken as broadly representative of all intersex people or all HCPs implicated in their care in Canada. Recruitment posters emphasized ‘improving’ practices of health communication and contained the language of ‘intersex’, which likely biased our HCP sample toward affirmative practitioners who believed changes need to occur. HCPs have been difficult to recruit for this subject in Canada [see (13)] and the knowledge and insights shared by our HCP interviewees were invaluable toward our research aims. At the same time, this research is missing perspectives from HCPs who may be more recalcitrant or want to solely work within a biomedical model. Finally, as our study design generated themes raised by and important to participants across interviews and groups, while centering the needs and lived experience of intersex participants, it precluded more in-depth focus on the HCPs in the study, and/or on a given theme.

Follow-up research and initiatives could work toward resourcing and implementing change along the three axes for improving intersex health communication identified in this paper. HCP health communication training and continuing education opportunities need to be developed, resourced, and implemented in consultation and collaboration with the intersex community (13). Affirmative HCPs could also be instrumental to intersex community efforts toward policy change in tracking and access to health data (12) and may be the most likely to reach those HCPs more reluctant to practice agency-based care. Issues of structural access barriers and gaps could benefit from general resourcing of intersex health services and supports at local, provincial and national levels (12, 13). A proactive approach is also needed to create and enact human rights protections for people with VSC in the medical arena (9, 12) awareness raising and sensitization efforts for the general public as well as HCPs would be helpful toward the naturalization and normalization of VSC (12). Further literature forefronting intersex lived experience, perspectives, and needs in healthcare in Canada is very much needed as there is a significant gap in the literature in this area. There were themes within this research that deserve more granular attention, such as the emotional and cognitive impacts of adverse health communication, including how people with VSC anticipate and have to manage stigma. Similarly, participants described practices of resilience and what they needed for healing and repair work. Fuller understanding of effective mental health support for intersex people is important and will enhance HCP education efforts (13). Future research could utilize a similar agency-based, social-ecological framework adapted to the project aims.

## Conclusion

Taken together, participant accounts in this study depict a landscape for intersex health communication in Canada where significant change is needed, but attainable. Interviews illustrated the resilience and agency of intersex participants, even within a healthcare system where they are systemically disenfranchised. They demonstrated considerable initiative from individual HCPs looking to help shift practices toward affirmative care. Our conceptual framework was critical toward an understanding of the current problem as multi-layered and multi-faceted and not merely a matter of individual actions or solutions. This study highlights the need for collaborative efforts across multiple sectors and by multiple stakeholders to drive meaningful change. There needs to be greater attention to and a lowering of healthcare access barriers for intersex people, promotion of accessible affirmative agency-based standards of care, ending of harmful medical practices, and fostering of greater societal awareness and inclusion. From an equity lens, Canada has an ethical imperative to provide better access to and quality of care for people with VSC. Positive policy, increased health services resourcing and explicit legal protections could go a long way toward realizing these goals. Ultimately for participants, affirmative health communication was not only about achieving a baseline of knowledge about VSC, or following a guide, but about being seen, heard, and supported in their individuality and humanity, to pursue their health and wellbeing, and to make the fully informed decisions that are the best for their lives. It was about a quality of heart, of presence, and of really caring.

## Data Availability

The datasets presented in this article are not readily available because interview transcripts cannot be fully anonymized and participants did not provide consent for the sharing of transcripts with parties other than the researchers. The complete codebook will be available upon request. Requests to access the codebook should be directed to TK, terese.knoppers@mcgill.ca.
