# Supplementary material for: “A quality of heart, of presence, and of really caring”: toward affirmative intersex health communication in Canada
Source: Front Public Health. 2025 Jan 6;12:1436354. doi: 10.3389/fpubh.2024.1436354 (PMC11743699; doi:10.3389/fpubh.2024.1436354)
Supplement: Supplementary file 1 [file Data_Sheet_1.docx]

**Appendix 1: Semi-structured interview guides**

*Note: These were the general topics covered, but as these were semi-structured interviews, the wording and order were not always the same, questions were adapted to the interviewee, and other questions were asked based on what participants brought up and what topics and concerns were most salient to them.*

***Questions for intersex* participants***

**use interviewee terminology throughout*

1. Do you have any questions for me or about the study? What interested you about this study?
2. How central is being intersex to you? How central is it to your everyday life?
3. How do you find the medical professionals that you go to? Do you visit the same healthcare practitioners on a regular basis? What are your hopes/expectations for your interactions with your healthcare practitioners?
4. Did you become aware of having an intersex variation through a medical professional? If so, how was this for you? Is there anything you would change about how it was communicated?   (e.g. was it clear, well-paced, was there room for you to ask questions, etc.)? Was there attention to your emotional world - your concerns and needs?
5. Have you had any experiences with healthcare practitioners that stand out as sensitive, appropriate, respectful and/or supportive?
6. What would you like to change about the interactions you have had with healthcare practitioners? Have you had experiences where you feel you were treated differently because of being intersex?
7. What is something you wish was in the general curriculum for healthcare practitioners learning to communicate with patients? What about health communication with individuals with intersex variations specifically?
8. Do you have any advice for intersex people on navigating the Canadian healthcare system?
9. Is there anything else that you would like to add?

***Questions for healthcare provider participants***

1. Do you have any questions for me or about the study? What interested you about this study?
2. What does your day-to-day healthcare practice look like? Can you describe your medical background/years in the field?
3. What kind of experience do you have with providing care to intersex patients?
4. Within your area/specialty, did you have training on working with patients with variations in sex characteristics? If so, can you describe it? Do you know of opportunities in your field for continuing education in this area?
5. In your medical practice what is important to you about your health communication with patients? Are there types of communication or information delivery to patients that you find particularly challenging? What sorts of tools do you use for respectful and sensitive health communication?
6. If you were to discover that a patient is intersex and might not be aware, how would you manage disclosure? What would you do to support your patient?
7. What are some medical or psychosocial concerns that may come up in the course of care provision for a patient who is intersex?
8. Do you have any input from your specific specialty/field that you would like to add?
9. Is there anything else that you would like to add?
